# Humans can find rhythm in randomly timed sounds

**DOI:** 10.1098/rsos.250453

**Published:** 2025-08-20

**Authors:** Jelle van der Werff, Tommaso Tufarelli, Laura Verga, Andrea Ravignani

**Affiliations:** ^1^ Department of Human Neurosciences, University of Rome La Sapienza, Rome, Lazio, Italy; ^2^ Comparative Bioacoustics Group, Max Planck Institute for Psycholinguistics, Nijmegen, Gelderland, The Netherlands; ^3^ Unaffiliated, Beeston NG9, UK; ^4^ Department of Neuropsychology and Psychopharmacology, Maastricht University, Maastricht, Limburg, The Netherlands; ^5^ Center for Music in the Brain, Department of Clinical Medicine, Aarhus University and The Royal Academy of Music Aarhus/Aalborg, Aarhus 8000, Denmark

**Keywords:** rhythm, timing, temporal randomness

## Abstract

Humans are keen pattern-seekers and take advantage of regularities present in their environment. In the temporal domain, we may call these patterns rhythms, but what is rhythm? Definitions vary, but all presuppose a categorical distinction between rhythm and randomness. Here, we challenge this view and show that two types of random sound sequences—classically considered arrhythmic by experimenters—differ in the amount of regularity humans reconstruct from them. When asked to synchronize to randomly timed sounds, participants leverage statistics to estimate the underlying tempo of the sequence, similar to linear statistical estimators. Theoretically, our results challenge current definitions of rhythm by showing that rhythmicity and randomness are instances of a continuum. Methodologically, our data and mathematical model show that a common method for creating random timing, namely the jittering of event onsets, introduces an undesirable regularity that humans readily exploit. New experiments should aim to maximize temporal randomness, and past experiments’ outcomes require reconsideration.

## 1. Introduction

**Figure 1.**
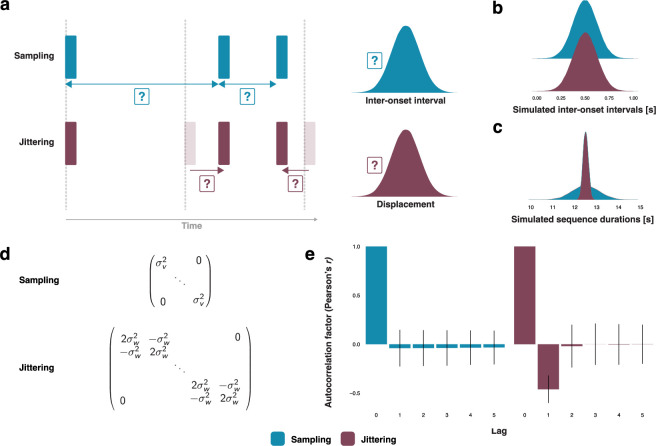
(a) Two example sequences randomly timed using the sampling and jittering methods, respectively. If *t* (tempo) represents the intended mean inter-onset intervals (IOI; i.e. *μ* of the sampling distribution), *v* represents the variance for the sampling method and *w* represents the variance for the jittering method (electronic supplementary material). The resulting IOIs of sampling sequences can be described as $IOI_{i}=t+v_{i}$, and for jittering sequences as $IOI_{i}=t+w_{i+1}-w_{i}$. Note that the sampling method can involve sampling IOIs as well as inter-stimulus intervals, i.e. offset-to-onset rather than onset-to-onset. For simplicity, we focus on IOIs, though all derivations are equally valid for inter-stimulus intervals. Similarly, while here we use continuous normal distributions, our mathematical formalisms identically apply to different sampling distributions (e.g. discrete uniform). (b) Probability distributions of IOIs for simulated sampling and jittering sequences (see §5). The IOIs’ distributions are statistically indistinguishable across methods (two-sample Kolmogorov–Smirnov *D* = 0.003, *p* = 0.553). (c) Probability distributions of the durations of simulated sequences randomly timed using the sampling or the jittering method. Sequences created using the jittering method are less variable in overall duration. (d) Covariance matrices showing dependencies between IOIs in sampling or jittering sequences. The sampling method’s covariance matrix has a diagonal structure, while the jittering method’s covariance matrix has a tridiagonal structure. (e) Autocorrelation factors (Pearson’s *r*) for IOIs of simulated sampling and jittering sequences. We find that for sampling, the IOIs are non-correlated (lag one: mean *r* = −0.04, s.d. = 0.19), whereas for jittering, they are negatively lag-one autocorrelated (lag one: mean *r* = −0.46, s.d. = 0.18). Error bars indicate mean ±  s.d.

To understand how humans process sub-second rhythmic regularities, one can compare temporal regularity with its converse: temporal randomness. Isochrony is the highest form of regularity; like a metronome or a ticking clock, isochronous events (e.g. sounds) are spaced evenly in time, resulting in a series of identical inter-onset intervals (IOIs). What constitutes temporal randomness instead? Commonly used in experiments, temporal randomness provides a control condition against which to compare the processing of rhythmic stimuli. In general, one of two methods is used to create random timing (figure 1a). Both result in indistinguishable distributions of IOIs (figure 1b), yet differ in other respects. The first method, which we named interval sampling (henceforth ‘sampling’), samples the time *intervals* between events from a distribution and concatenates them. The total duration of a sequence equals the sum of the sampled IOIs and can vary. The second method, which we named onset jittering (henceforth ‘jittering’), displaces the *events* in an initially isochronous sequence by values sampled from a distribution. With this method, the total duration of a sequence is less variable (figure 1c), and we suspect this to be the reason this method is frequently used.

The simplest possible example to illustrate the difference between the two methods to create random timing is a sequence of three events and therefore two IOIs. An isochronous sequence of this kind could feature three events separated by two IOIs of 1 s. To obtain the sequence’s random counterpart using the jittering method, one would displace the second onset by a random amount, say −0.5 s, hence generating adjacent IOIs of 0.5 and 1.5 s. To obtain the sequence’s second random counterpart, obtained via the sampling method, one would simply generate two IOIs sampled from a distribution of e.g. 1 and 1.5 s, respectively. Notice how the two IOIs are statistically independent in the sampling case but not in the jittering case. When jittering, a decrease of the first IOI by 0.5 s results in an increase of the second IOI by the same 0.5 s. Though it is difficult to estimate the prevalence of jittering, we have identified its use in the sub-second range for randomly timing stimulus onsets in behavioural and EEG experiments (e.g. [1–6]), but also in the timing of TMS pulses (e.g. [7–9]).

## 2. Human sensitivity to different random timing methods

Until now, sampling and jittering have been treated as equivalent methods for producing random temporal sequences. However, we observe a fundamental difference when comparing the structures of their respective covariance matrices (figure 1d). In sampling sequences, consecutive intervals are non-correlated. In jittering sequences, consecutive intervals are negatively lag-one autocorrelated (figure 1e). Short-range correlations like these are known to occur, for instance, in the rhythmic structure of conversation [10], and between taps in sensorimotor synchronization experiments [11]. To date, no research has unequivocally demonstrated a perceptual advantage for autocorrelations during sensorimotor synchronization (cf. [12,13]). Such a perceptual advantage would be a confound for experiments in which temporal regularity is contrasted with temporal randomness. In these cases, type II errors can emerge when regular and random conditions are less perceptually divergent than assumed.

Here, we compare sampling sequences with jittering sequences using a three-pronged approach: First, we behaviourally test whether the autocorrelated intervals that result from using the jittering method allow listeners to more readily synchronize with these arrhythmic stimuli. Second, we provide closed-form mathematical formalisms to show the influence of correlation structures on rhythmic tempo estimation (electronic supplementary material). Finally, we compare our behavioural results with mathematical simulations of tempo estimation, showing how different estimation strategies compare with our participants’ behavioural performance. We provide mathematical formalisms and code for running our simulations (see Data accessibility) to facilitate *a priori* analyses of random timing conditions in future experiments when different parameters are used from those used here.

**Figure 2.**
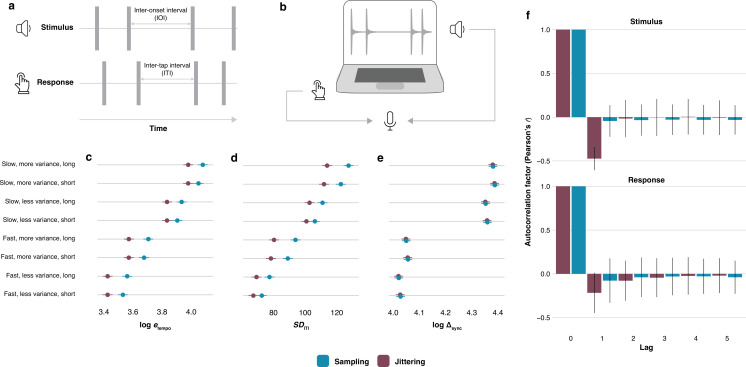
(a) In an online finger-tapping experiment, participants (*n* = 135) tap along with sequences of pure tone sounds, randomly timed using the jittering or sampling method. Sequences differ in tempo, in the amount of variance used in the sampling distributions, and in length (see §5). (b) Responses are recorded together with the stimulus using the microphone of the participants’ laptops, which ensures accurate recording timing. They are subsequently analysed using the software REPP [15]. (c) Estimated marginal means for $\log e_{tempo}$, calculated as the log-transformed absolute difference between each inter-tap interval (ITI) and the tempo of the stimulus sequence. $\log e_{tempo}$ is lower for jittering sequences than for sampling sequences (mixed-effects model: *t*(185 622) = −10.37; *β* = −0.09, 95% CI: −0.10, −0.07; *p* < 0.001). (d) Estimated marginal means for *SD*_ITI_, calculated as the standard deviation of the ITIs within each sequence. Tapping variability is lower for jittering sequences than for sampling sequences (mixed-effects model: *t*(9823) = −4.70; *β* = −0.13, 95% CI: −0.18, −0.08; *p* < 0.001). (e) Estimated marginal means for log⁡ Δsync, calculated as the log-transformed absolute difference between each response onset and the corresponding stimulus onset. There are no differences in stimulus–response asynchronies between conditions (mixed-effects model: *t*(224 895) =ITI −0.09; *β* = 0.00, 95% CI: −0.01, 0.01; *p* = 0.931) and (f) autocorrelation factors (Pearson’s *r*) for the stimulus IOIs and response ITIs in the sampling and jittering conditions, collapsed across the different tempi, degrees of variance and sequence lengths. Error bars indicate ± s.d. Lag-one autocorrelations in participants’ responses are higher for jittering (*M* = −0.22, s.d. = 0.23) than for sampling (*M* = −0.08, s.d. = 0.25, mixed-effects model: *t*(9808) = −30.00; *β* = −0.56, CI: −0.59, 0.52; *p* < 0.001). For (c–e), error bars indicate 95% CI.

We use an established finger-tapping paradigm [14] to compare human synchronization performance in response to sequences of beeps randomly timed using either the sampling or the jittering method (figure 2a,b and electronic supplementary material, figure S1). Based on previous work, we assume that all presented sequences are perceived by the participants as too irregular for synchronization [16,17]. We hypothesize that, as a result of the difference in correlation structures between sampling and jittering, participants will perform better when tapping to jittering—as opposed to sampling—sequences on three dependent variables. First, we expect participants to more accurately estimate the underlying sequence tempo for jittering sequences. This would show as lower $e_{tempo}$: a smaller absolute difference between the response inter-tap intervals (ITIs) and the mean of the sampling distribution. Note that the term ‘tempo’ is used loosely throughout this manuscript, referring simply to a sequence’s expected average IOI (as per the formulae in the caption to figure 1a). Second, we expect more regular, less variable tapping [18] in response to jittering sequences (lower *SD*_ITI_). Third, we expect higher tapping accuracy in response to jittering sequences, resulting in smaller stimulus–response asynchronies ($\Delta sync\;=\;|{onset}_{stim}-{onset}_{resp}|$). In addition, we expect a preservation of short-range correlations from stimulus to response for jittering sequences (cf. [12]).

As predicted, participants tap closer to the stimulus tempo in response to jittering sequences compared with sampling sequences (figure 2c). They also tap more regularly (figure 2d). However, they do not tap more accurately (figure 2e). Together, these three results suggest a tapping strategy of regularization [19], facilitated by lag-one autocorrelated intervals in jittering sequences. The lag-one autocorrelation in jittering stimulus sequences is *negative*, meaning that on average, jittering sequences have a pattern of alternated short–long intervals. If participants choose to tap such a pattern, stimulus–response asynchronies would be smaller for jittering sequences compared with sampling sequences. This does not seem to be the case: rather than aiming to minimize asynchronies, participants tap more regularly by estimating the underlying sequence tempo. This inclination towards regularization also explains why previous research did not demonstrate an effect of short-range correlations on stimulus–response asynchronies [12,13]. Also consistent with these studies, our results show a preservation of short-range correlations from stimulus to response for jittering sequences (figure 2f).

## 3. Mathematical model of tempo estimation

**Figure 3.**
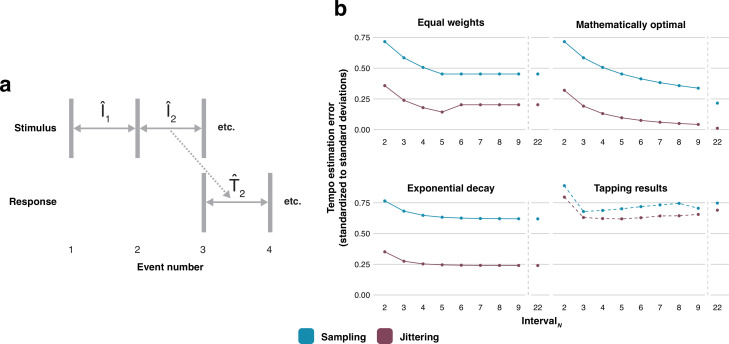
(a) The first estimation of tempo in the finger-tapping experiment is the interval between events three and four (as participants are instructed to start tapping from the third sound onwards, see §5). For both the model and the tapping results, we name the first estimated tempo value ${\hat{T}}_{2}$, which maps to the stimulus interval that was last encountered, i.e. ${\hat{I}}_{2}$ (electronic supplementary material). (b) Modelled and measured tempo estimation error ($e_{tempo}$), standardized to standard deviations of the sampling distributions (see §3). Lower values mean better tempo estimation. The final interval shown (interval 22) is the last interval that was perturbed in all experimental conditions, occurring between onsets 23 and 24. The tapping results depict the differences in tempo estimation error between the sampling and jittering methods, collapsed across all other conditions. The tapping results compared with the model’s results show an additional constant error (i.e. both lines are shifted up for the tapping results) and tempo drift (i.e. the amount of error increases as the sequence progresses).

Our data show that listeners can better estimate a sequence’s underlying tempo based on lag-one autocorrelated intervals (jittering) compared with non-correlated intervals (sampling). We present a mathematical model (figure 3 and electronic supplementary material) to understand why this is the case, taking into account different scenarios for how these intervals are stored in working memory when estimating a sequence’s tempo. The model can be adapted to describe sequences with arbitrary correlation structures. Here we use it to compare negatively lag-one autocorrelated (jittering) intervals to non-correlated (sampling) intervals. We compare three possible estimation strategies: (i) equal weights (importance) for all intervals, with a ‘memory cut-off’ after five intervals (cf. [20–22]), (ii) mathematically optimal weights with no memory cut-off (infinite memory), and (iii) exponentially decaying weights [23–25], where there is no memory cut-off but each newly experienced interval outweighs the previous one by a constant factor, i.e. a recency effect. For the latter strategy, we assume a half-life of 1 s [26]. The exponential decay strategy may be considered the most realistic: in general, human cognition shows recency effects, and flexible synchronization [27] in particular demands recently encountered intervals to be weighted more heavily than intervals encountered earlier [28,29].

Our model and experimental results (figure 3) reveal that for all the estimation strategies considered here, the tempo of jittering sequences is easier to estimate than the tempo of sampling sequences. This difference occurs early during the presentation of a sequence and remains of similar size throughout. For the equal weights strategy, longer sequences result in better tempo estimations until the number of encountered intervals (*N*) reaches the memory cut-off after five intervals, at which point the estimation accuracy plateaus (cf. [25]). The mathematically optimal estimation strategy provides a tempo estimation precision that improves indefinitely with larger *N*. Importantly, this strategy allows for tempo estimates that improve with larger *N* faster for jittering than for sampling (electronic supplementary material). We only modelled tempo estimation strategies, and so the model does not consider factors such as motor variability, error correction and tempo drift [14,18]. From the model, it follows that the differences in estimation accuracy between the sampling and jittering methods are not dependent on the number of intervals in the stimulus sequence, nor on the distribution of weights. In all scenarios, tempo can be estimated more accurately for jittering sequences than for sampling sequences, and this difference materializes quickly and consistently, analogous to our behavioural results.

## 4. Discussion

Tempo estimation is core to rhythm perception. With no perceived tempo or pulse, musicians would not be able to make music together, and listeners would not be able to synchronize. The subjective perception of a regular underlying pulse is therefore a defining property of rhythm [23]. Pulse extraction is also a prerequisite for subsequent (hierarchical) pattern processing, such as during music perception [23], and interacts with pattern-seeking and statistical learning processes through attention [30]. In rhythm experiments, it is assumed that once surpassing a certain irregularity threshold [17,31], the listener will perceive a sequence of sounds as temporally random. Accordingly, we expected all presented sequences to be perceived as equally random because our stimuli surpassed these thresholds (see §5). In future experiments, explicit judgement tasks can shed light on whether all sequences were indeed consciously perceived as equally random by the participants. Similarly, here we chose to only use simple sounds with a clear perceptual onset. For now, it remains unclear how our findings would generalize to more complex sounds, such as in speech, where perceptual onsets and perceptual centres can differ from sound to sound, but also from listener to listener [32]. Finally, in future work, individual differences will have to be more extensively studied. Here, we chose to include participants as a random factor in our models, and so while revealing a general trend, the extent to which individual differences contribute to the reported effects remains unclear.

We show that when listeners are presented with different types of sequences that are subjectively all highly arrhythmic, the underlying pulse (or tempo) is nonetheless estimated. Statistical properties resulting from methodological choices then determine the accuracy of those estimations. In order to flexibly synchronize [27] with tempo changes, listeners naturally take into account only a limited number of intervals when estimating tempo. Our behavioural and modelling work shows that differences in statistical structure can increase the accuracy of the tempo estimation after encountering only two intervals. It may seem surprising that perceptual benefits resulting from differences in statistical structures emerge so quickly. However, similar statistically dependent mechanisms for non-periodic timing probably underlie many fast perceptual processes, as recently suggested for speech (e.g. [10]), and for the processing of naturalistic sounds (e.g. [33,34]). Humans readily exploit statistics to improve timing estimates, and so, to fully avoid synchronization or entrainment artefacts, control conditions in rhythm experiments must be sufficiently random (cf. [16,31]). Methodologically, from our study, we conclude that this is not the case when stimulus onsets are jittered. Interval sampling is the better—more random—choice.

Our study showcases the extent of the human propensity for regularity extraction. Temporal regularities are extracted even in cases where that regularity is non-obvious. Then what is temporal randomness? Based on our study, we conclude that temporal randomness is not a single categorical entity on the opposite side of regularity and rhythmicity, but that it can be part of the same continuum. Our results demand a careful reconsideration of how temporal randomness is used in experiments and suggest a closer relationship between regularity, rhythm and randomness than previously assumed.

## 5. Methods

### 5.1. Ethical statement

All procedures adhered to the ethical standards of the Ethics Committee Social Sciences of Radboud University (agreement number ECSW-2021-068). Participant data were handled according to the General Data Protection Regulation (GDPR) and Max Planck Society regulations. Informed consent was obtained from all participants before participation. Participants received monetary compensation.

### 5.2. Sampling distributions for random timing methods

Two types of sequences were used in both the finger-tapping experiment and in the simulations (e.g. figure 1): (i) sequences created using the sampling random timing method and (ii) sequences created using the jittering random timing method. For the sampling method, the IOIs were *sampled* from a normal distribution and concatenated. For the jittering method, the event onsets of completely regularly timed (isochronous) sequences were *displaced* by values taken from a normal distribution. Different standard deviations were used for sampling IOIs (for the sampling method) and displacements (for the jittering method). This is necessary to ensure identical distributions of the IOIs in both types of sequences. Indeed, from the methods’ formulae (figure 1), it can be derived that the relationship between the variances of the IOIs for the sampling method compared to the jittering method is


(5.1) $$\sigma_{v}^{2}{=}_{\;}2\sigma_{w}^{2},$$

where ${\sigma_{v}}^{2}$ represents the variance of the IOIs for the sampling method, and ${\sigma_{w}}^{2}$ represents the variance of the IOIs for the jittering method. Consequently, the standard deviations used for the displacements of jittered sequences were calculated as


(5.2) $$\sigma_{w}\;=\;\frac{\sigma_{v}}{\sqrt{2}}.$$

The standard deviations used were additionally dependent on sequence tempo (see §5.4). For sampling, the final standard deviations were calculated by multiplying one of two arbitrary values (0.2 or 0.25) by the sequence tempo (400 or 500 ms), resulting in four different standard deviations. These standard deviations were then entered into equation (5.2), resulting in the four standard deviations used for the jittering method (electronic supplementary material, table S1).

### 5.3. Simulations

To simulate the distributional properties of sampling and jittering sequences, we generated 10 000 random sequences for both types (*n* = 20 000). Each sequence contained 25 IOIs. Sequences were generated using a value for the intended average IOI of the sequence (*t*) of 500 ms, and standard deviations of 125 ms (sampling) and 88.38 ms (jittering).

### 5.4. Design

The finger-tapping experiment was conducted online using the participant’s personal laptop. Online finger-tapping experiments have recently become a reliable method for sensorimotor synchronization research thanks to the REPP software package [15]. REPP is a Python package that performs signal processing to analyse recordings of finger-tapping responses. REPP avoids the common stimulus–response timing inaccuracies of online experiments by using the internal microphone of a participant’s laptop to record a participant’s taps together with the original stimulus played back through the laptop’s speakers (figure 2b). Unlike most laboratory experiments, participants tap directly on the casing of their laptops, and the timing of this response is analysed in relation to the timing of the stimulus (electronic supplementary material, figure S1).

The experiment followed a classic synchronization–continuation paradigm (e.g. [35]). During the *synchronization phase*, participants tapped while listening to the sound sequences. In the *continuation phase*, after the final sound, participants continued tapping for another four intervals while no stimulus was presented. The experiment had a 2 × 2 × 2 × 2 design. A trial either: (i) was constructed using the sampling or jittering random timing method, (ii) had a sequence length of 25 or 35 sounds, with an additional five virtual taps in the continuation phase, (iii) had IOIs or displacements sampled from a normal distribution with a small or a large amount of variance, and (iv) was presented either at a slow (mean IOI = 500 ms) or a fast tempo (mean IOI = 400 ms). Five trials were presented for each of the 16 combinations, with a total number of 80 trials presented over eight blocks. The order of trial presentation was randomized for each participant. We included manipulations of tempo, sequence length and variance to improve the generalizability of our results across sequences of different tempo, length and generated using sampling distributions with different amounts of variance, as might be encountered in different rhythm experiments.

### 5.5. Stimulus

The stimulus in each trial was a 1000 Hz sine wave of 50 ms, with a 5 ms linear on- and off-ramp. A bell sound indicated the end of each trial. Because of the online set-up, it was not possible to control the playback volume directly. However, before starting the experiment, participants could adjust the speaker volume using an example sound and a sound meter graphical interface.

For all trials, the first sound was used to define the initial time t=0. The final sound was placed at the earliest possible isochronous position following the sequence’s penultimate sound (electronic supplementary material, figures S1 and S2). We included such an event, as well as the ensuing continuation phase, because in some experimental set-ups the final event in a sequence is not perturbed (e.g. [1,36]). Theoretically, such placement allows calculating the exact sequence tempo on the basis of all intervals in a sequence (i.e. $tempo=\frac{t_{n}-t_{0}}{n}$) [28]. However, no such effect was observable (electronic supplementary material, figure S2), which is why for all analyses only the responses from the synchronization phase were considered.

We included sequences of two lengths, namely 25 or 35 events. We manipulated sequence length following initial calculations that showed that the accuracy of tempo estimations for jittered—but not for sampling—sequences would increase with a larger number of experienced intervals. We expected an interaction effect between random timing method (sampling versus jittering) and the number of intervals in a sequence on two of our three dependent variables, for which we found some evidence ($e_{tempo}$ and *SD*_ITI_; electronic supplementary material, figure S3).

Sequences had one of two tempi; the normal distributions used to sample IOIs (sampling) or displacements (jittering) had a mean of 400 or 500 ms. These values were chosen such that few IOIs were shorter than 200 ms [14] and so values were in the neighbourhood of preferred spontaneous tapping tempi, which have been suggested to lie at around 500 ms [37].

We included two different degrees of variance for the sequences (i) to minimize the frequency of overlapping events—which would occur when the IOIs were shorter than the duration of the sounds, i.e. 50 ms, (ii) to maximize the number of supraliminal [17] displacements, and (iii) to maximize the distance between standard deviations in the two variance conditions. In cases where the resulting IOIs led to overlapping events (i.e. IOIs shorter than the event duration of 50 ms), we sampled the sequence again. This changed the shape of the used sampling distributions slightly, though with negligible differences: only 19 sequences were re-sampled out of the 10 800 sequences in the final dataset.

### 5.6. Procedure

A custom website was designed for the online finger-tapping experiment. The experiment consisted of a training phase and a testing phase. Before starting the training phase, participants’ laptop set-ups were tested using an automated analysis to ensure that the recordings were audible and did not contain background noise. This test also checked whether there were timing inaccuracies in the presentation of the test sounds, which can occur in cases of high computer memory load. If the automated system check failed, participants received feedback on possible solutions, such as changing the speaker volume or eliminating background noise. In cases where the system check failed three times, participants were automatically excluded.

Participants were asked to provide their age, gender and handedness (factors known to potentially affect tapping performance) [38–40], as well as their first language. In addition, they filled out a questionnaire consisting of eight questions about their musical experience.

After reading the instructions, participants started a training phase, which consisted of four trials. The training phase always consisted of the same four stimulus sequences (electronic supplementary material, table S2). Participants were allowed a maximum of three rounds of training. During the training phase, participants were given feedback after each trial if too few taps were recorded in either the synchronization phase (fewer than 70% of the number of stimulus events) or the continuation phase (fewer than three out of five), or when the calibration sounds necessary for the response analysis were inaudible. Participants directly continued to the testing phase after three or more correct trials in any of the rounds.

Participants received as little instruction about the task as possible to avoid inducing any particular tapping strategy. They were asked: ‘Tap along with the beeps as best you can, starting from the third beep you hear, and do not stop until you hear the bell’. Participants were instructed to tap using their index finger. It is common practice to ask participants to start tapping from the third event onwards (e.g. [35]) because at least two IOIs are necessary for making an estimation of when the next event will occur. During the continuation phase, no sounds were presented to the participant.

In the experiment phase, no feedback was provided to the participant. The experiment consisted of eight blocks of 10 trials. Between blocks, participants were allowed to take a break. Each of the eight blocks lasted around 3 min. The summed total of all stimulus durations was around 30 min. The total experiment duration was around 45 min.

### 5.7. Pilot study

A pilot study was conducted (*n* = 10) prior to starting data collection for the finger-tapping experiment, mostly to test our experimental set-up. One participant was excluded because of non-compliance (final *n* = 9). Based on this preliminary investigation, two aspects of the experimental procedure were changed. First, the number of rounds for the training phase was increased from two to three. Second, the training instructions were modified to clarify that five taps were required after the synchronization phase of the experiment. To avoid ‘data peeking’ [41,42], the pilot study data were only checked for completeness, but not analysed. In our analyses, including pilot versus non-pilot as a factor did not significantly improve model fit. Therefore, and since no changes were made to any aspect of the experimental procedure itself, the pilot study’s results were pooled together with those from the main experiment.

### 5.8. Participants

Data were collected in the autumn of 2021. One hundred and fifty participants were recruited through the subject database of the Max Planck Institute for Psycholinguistics. Ten out of these 150 participants were excluded either because of technical issues (e.g. microphone failures) or because they could not complete the training phase of the experiment. The main technical issues were microphone issues, which were detected by the automated system check that preceded the experiment. Another five participants (including one from the pilot study) were excluded because both the automated response analysis and the follow-up manual inspection detected more taps than expected (i.e. more than 150% of the number of events in a sequence).

The final sample (including the pilot participants) consisted of 135 participants (104 females; $M_{age}$= 29 years, $s.d._{age}$= 13, age range: 18–75). There were 13 self-reported left-handed and two ambidextrous participants.

### 5.9. Analysis

The response analysis was performed using REPP [15]. Based on the calibration sounds at the beginning and end of the response recordings, REPP calculated the onsets of the stimuli and of the response taps. A number of failing criteria determined whether a trial was marked as correct or incorrect: the number of tapping responses needed to be higher than 70% of the number of presented events to ensure a response was made. The number of responses also needed to be lower than 150% of the number of presented events to ensure participants did not tap irrespective of presented stimuli. Finally, the three calibration sounds presented at the beginning and end of a trial were required to be audible.

In order for REPP to associate a particular tap with an event, responses needed to be within a phase difference window of −0.4 to 0.4. Here, the period preceding the stimulus onset would run from −1 to 0, and the period following the stimulus onset would run from 0 to 1. Taps outside of these values were not considered in the rest of the analyses and were considered ‘missing’. REPP calculates phase differences as follows: the onset of each event is subtracted from the onset of a tap. If the resulting value is negative, that value is divided by the closest stimulus IOI that precedes the event. If the resulting value is positive, it is divided by the closest stimulus IOI that follows the event.

In total, our raw data contain 10 800 trials; however, one trial was not recorded by the experiment website due to a failure in the response database. In addition, trials meeting one of the failing criteria were discarded (7.71% of 10 799 trials; electronic supplementary material, table S3).

Missing taps were removed (electronic supplementary material, table S4), as well as taps made in response to the first two events or to the final presented sound. For calculating ITIs, two consecutive non-missing taps are required. The analyses using ITIs, therefore, use a smaller sample. In general, because these sequences were highly irregular, a relatively large amount of missing data was expected.

All analyses were performed in R [43]. The used code, including raw and processed data, as well as statistical models and their results, is available from GitHub (see Data accessibility). Because tapping data is particularly influenced by individual differences [44], we analysed the data using a linear mixed-effects model with participant as a random factor. Three models, one for each dependent variable, were built by comparing model fit using the *anova.lme* function from the *nlme* package [45]. At each step, additional factors were only kept if model fit improved significantly (*p* < 0.05). For each of the three models, we started from a baseline model that included only the (fixed) intercept and progressively added random effects for participants, the random slopes, the different predictors and, finally, expected interaction effects. Predictors were entered in this order: sequence tempo, amount of variance in the sequence, random timing method (sampling or jittering), sequence length and participant’s age, handedness and gender. Because of the large sample size, no normality tests were carried out. However, histograms for each dependent variable are presented in electronic supplementary material, figures S3–S5. Dependent variables $e_{tempo}$ and Δsync were log-transformed to mitigate their distributions’ skew, which was the result of taking absolute values.

### 5.10. Mathematical model

The mathematical model (figure 3 and electronic supplementary material) assumes that humans behave like linear, unbiased statistical estimators (cf. [28,46]). Participants are assumed to perform a weighted average of *N* elapsed IOIs to estimate the underlying period. It is further assumed that participants are able to provide the correct estimation *on average*. Within this framework, an estimation strategy is uniquely defined by a vector of weights **w**, whose components specify the importance of each experienced IOI in influencing the current estimation of the underlying period. The model then predicts that the estimation error depends on the chosen weights as well as on the covariance matrix of the IOIs. The latter is a matrix that encodes the variances of each individual IOI as well as the statistical correlations between them. To compare model predictions and experimental data, we use ITIs as a proxy for a participant’s estimate of the underlying period. This means that the very first period estimate (i.e. the estimate that informs the timing of the very first ‘tap’) is not experimentally observable.

## Data Availability

Data and relevant code for this research work are stored in GitHub [47] and have been archived within the Zenodo repository [48]. Supplementary material is available online [49].
